# Evaluating In-Car Movements in the Design of Mindful Commute Interventions: Exploratory Study

**DOI:** 10.2196/jmir.6983

**Published:** 2017-12-04

**Authors:** Pablo Enrique Paredes, Nur Al-Huda Hamdan, Dav Clark, Carrie Cai, Wendy Ju, James A Landay

**Affiliations:** ^1^ Human Computer Interaction Group Department of Computer Science Stanford University Stanford, CA United States; ^2^ Interaction Design Research Center for Design Research Stanford University Stanford, CA United States; ^3^ Media Computing and Human-Computer Interaction Group Rheinisch-Westfälische Technische Hochschule Aachen University Aachen Germany; ^4^ Glass Bead Labs Baltimore, MD United States; ^5^ Computer Science and Artificial Intelligence Lab Massachusetts Institute of Technology Cambridge, MA United States; ^6^ Information Science Cornell Tech New York, NY United States

**Keywords:** mental health, stress, stress management, mindfulness, in-car experience, interventions, just-in-time interventions, autonomous vehicles, cars, driving, breathing, mindful movement

## Abstract

**Background:**

The daily commute could be a right moment to teach drivers to use movement or breath towards improving their mental health. Long commutes, the relevance of transitioning from home to work, and vice versa and the privacy of commuting by car make the commute an ideal scenario and time to perform mindful exercises safely. Whereas driving safety is paramount, mindful exercises might help commuters decrease their daily stress while staying alert. Increasing vehicle automation may present new opportunities but also new challenges.

**Objective:**

This study aimed to explore the design space for movement-based mindful interventions for commuters. We used qualitative analysis of simulated driving experiences in combination with simple movements to obtain key design insights.

**Methods:**

We performed a semistructured viability assessment in 2 parts. First, a think-aloud technique was used to obtain information about a driving task. Drivers (N=12) were given simple instructions to complete movements (configural or breath-based) while engaged in either simple (highway) or complex (city) simulated urban driving tasks using autonomous and manual driving modes. Then, we performed a matching exercise where participants could experience vibrotactile patterns from the back of the car seat and map them to the prior movements.

**Results:**

We report a summary of individual perceptions concerning different movements and vibrotactile patterns. Beside describing situations within a drive when it may be more likely to perform movement-based interventions, we also describe movements that may interfere with driving and those that may complement it well. Furthermore, we identify movements that could be conducive to a more relaxing commute and describe vibrotactile patterns that could guide such movements and exercises. We discuss implications for design such as the influence of driving modality on the adoption of movement, need for personal customization, the influence that social perception has on participants, and the potential role of prior awareness of mindful techniques in the adoption of new movement-based interventions.

**Conclusions:**

This exploratory study provides insights into which types of movements could be better suited to design mindful interventions to reduce stress for commuters, when to encourage such movements, and how best to guide them using noninvasive haptic stimuli embedded in the car seat.

## Introduction

### Overview

Stress affects people worldwide [1,2], yet opportunities to engage people in improving their stress management and coping skills are scarce, in part, because of lack of time and lack of appropriate spaces [3]. Commuting, which in the United States consumes roughly 1 hour per day [4], presents itself as a unique opportunity to deal with stress. Commute offers both a window of time and a dedicated space for the use of stress management interventions. We believe that mindful commute technology can offer a unique opportunity to embrace this problem. Prior research has shown people’s preference for short movement-based (somatic) [5-8] and breathing interventions [9-12]. Behavioral interventions should be carefully designed to complement the cognitive and emotional demands of driving. Additionally, a side effect of movement is a potentially higher level of alertness, which could be beneficial for both driving performance and mental health.

Whereas modern seat design may tend toward finding usability solutions, ergonomics can be complemented with somatic or mindful approaches (see Cranz [13] for a thorough introduction). Mindful movement practices have been shown to benefit systemic wellness and mental health (see Clark et al [14] for a review). In this paper, we focus primarily on understanding how people respond to performing basic movements in the context of a typical car seat. We complement our analysis with an assessment of nonintrusive and nonverbal guidance to perform these movements. As a case study, we chose a simple set of interactions implemented on an array of actuators embedded in the back of the car seat. This investigation of basic movements and simple interactions lays the groundwork for future design of in-car mindful movement interventions. Additionally, it addresses the question of how an individual’s movement in the car—be it configural or breath-based—might contribute to improving their well-being.

We performed a semistructured viability assessment to gain insights on the use of a car as the scenario for the design of ecologically valid mindful movement interventions. We divided our study in 2 parts: (1) an exploration of the effect of basic movements on people’s perceived stress and driving performance and (2) the response to movement cues generated by vibrotactile haptic stimuli embedded in the car seat. In the first part, we explored a series of basic movements aimed at activating different parts of the torso, shoulders, head, and hips. We chose 12 movements that occur in practices that leverage mindful body dynamics, posture, and breathing. Some examples of such therapies are Hatha yoga [7] and yogic breathing [9,15]. Furthermore, alternative therapies, such as Feldenkrais [16] and the Alexander technique [13], leverage similar fundamental building blocks. We define perceived stress as the self-reported level of stress and perceived driving performance as a self-reported account of the difficulty of continuing driving while executing the movements. We correct all self-report metrics for individual differences by subtracting against an individual baseline and normalizing repeated measures.

**Figure 1:**
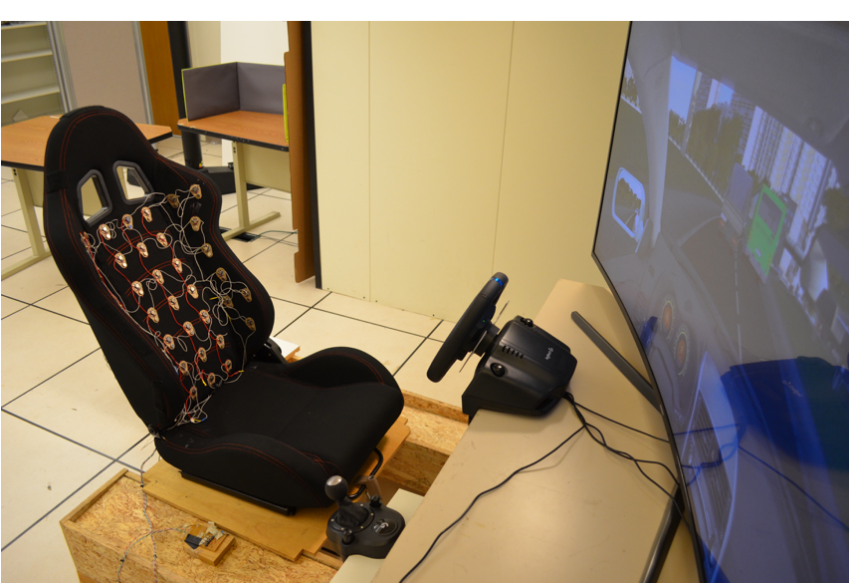
Vibrotactile prototype with a matrix of vibration motors in a car driving simulator setup.

We present insights drawn from participants driving under highway (simple) and city (complex) scenarios and using manual and autonomous vehicles. The second part of the study focuses on obtaining insights on the conceptual model and emotional response to vibrotactile patterns triggered by a haptic seat. The seat interface is made of a matrix of vibrotactile actuators (see Figure 1). Haptic patterns can be made by coordinating and combining individual actuators. Using the seat interface, we prototyped a range of patterns aimed at eliciting different basic movements. We discuss the adoption and usability issues of in-car haptic-guided movement.

### Background

#### Stress Management Interventions

While driving, constant attention on the road is stressful, although it makes us better drivers [17]. As a matter of fact, the Yerkes-Dodson inverted-U relationship between arousal and performance [18] shows that there is an optimal arousal level conducive to higher performance. Too much or too little arousal drives diminishing results. Stress at work can be described as a high arousal level, many times linked to solving challenges or facing threats [19]. With mindfulness training, stress could be reduced by focusing attention on the present.

In a car, we can implement technology that helps balance stress and attention [20]. Multiple sensors and actuators could be placed in proximity to the participant to sense and learn from affect [21] and reduce cognitive load [22]. Beyond traditional psychophysiology sensors [23], the car could also sense affect through voice [24], movement [25], pressure [26], or breathing patterns [27]. Furthermore, effective stress technology interventions [5,28] could be enhanced using multimodal actuation [29] and leverage entertainment systems to increase engagement [20].

It is important to take into consideration the contextual elements that can transform effective interventions in stressors. For example, lack of time, social contexts, or simply lack of concentration can reduce the efficacy of stress interventions [28,30]. New technologies for positive behavior change [31], as well as novel stress management interventions [32], could be repurposed for the car.

#### Ergonomic and Mindful Movement

Cranz [13] challenges the way we have been using chairs and seats as a passive instrument. She discusses how ergonomic research focuses most of its efforts to adapt our environments to our bodies and tasks. She argues that we should design environments that support our body’s natural inclination to move. Several studies have shown the physiological and mental gains obtained by engaging in movement in the office, even if it is just during breaks or intervals [33,34]. Complementary to this compulsory desire to move, Clark et al [14] describe the opportunity to use body movements as a way to engage the mind as well. They elaborate on the importance of understanding how a somatic approach to mindfulness takes advantage of the intrinsic value of the body as a conduit for awareness of the self and the environment. As described by Clark et al [14], the types of movements required to reach such levels of awareness and mindfulness are not necessarily high impact or strenuous. Quite the opposite is true; mindful movement often employs slow engagement of the body in repetitive patterns to allow for awareness of previously ignored but potentially high-impact differences. We propose that the opportunity to engage in mindful movement can be extensive when one is in a car, especially during the commute.

#### The Commute as an Ideal Scenario for Mindfulness

Mindful commute technologies could have a 2-fold impact. On 1 hand, mindfulness supports self-regulation and self-compassion [15], and research has shown that even a few minutes a day can reduce social stress impact [35]. On the other hand, brief and regular mindful interventions can improve attention control [33], which in turn could improve driving skills. Furthermore, in-car commuters can take advantage of 3 special characteristics of the commute: quietness, privacy, and relevancy. The car is a quiet and consistent space, which is the preferred setup for mindful practice [34]. The car is a private yet portable space, rolling at 60 miles per hour; no human beings can walk to the door. The time of day when the commute happens is relevant in terms of mental health and stress management. For example, commuters returning home often times carry with them residual stress from constantly facing and solving challenges at work [36]. Alternatively, commuters on the way to work may experience anticipatory stress [36,37] associated with high expectations of productivity. The commute presents not only an opportunistic but an ideal scenario and time to foster mindful behaviors. We propose that engaging in regular and brief mindful movements can benefit people’s health, improve their driving skills, and improve their perception of quality of living.

**Table 1:** Morphological box analysis of the design of in-car mindful movement interventions.

	Availability while driving	Relaxation potential	Movement range	Sensing potential	Actuation potential	Score (#highs/#lows)
Neck	High	High	High	Low	Low	1.5
Shoulders	High	High	High	Low	Low	1.5
Back	High	Low	Low	High	High	1.5
Hips	High	Low	Low	High	High	1.5
Lower extremities	Low	High	Low	High	Low	0.67
Upper extremities	Low	High	Low	High	Low	0.67

### System Design

#### Body Movements

In this paper, we focus our attention mainly on configural and breath-based movements, which we call breathing exercises in the rest of the paper. For the former, we observe movements that engage key musculoskeletal regions such as the neck, shoulders, back, and hips. For the latter, we draw inspiration from yogic breathing [9,15], which describes posture and breathing exercises aimed at reaching higher levels of awareness. We complement our exploration by observing movements used in 2 alternative techniques: Feldenkrais [16], which aims at mindful comparison of variations on intentional movements, and the Alexander technique [13], which leverages guided visualizations that are conducive to posture improvement [14,36].

To choose the parts of the body to engage in mindful movement, we performed a simple morphological box analysis [38] (see Table 1). We compared key parameters necessary to design mindful movement interventions versus large musculoskeletal groups and performed a simple binary ranking on each parameter (high vs low).

It is easy to recognize that extremities are highly engaged while driving, but other musculoskeletal regions are less engaged. Therefore, extremities are less available to be engaged in movement-based exercises. Hips and back seem to have a lower potential for relaxation. Neck, shoulders, and extremities all get activated when a fight-or-flight stress condition occurs [2]. Neck and shoulders have a higher movement range because of their smaller size and their relative freedom (ie, not touching any car component). This same freedom makes them difficult to sense and actuate on. Body parts in touch with the car could have a sensor or actuator placed in their location. Although hands and feet have direct contact with the car, other parts of the extremities are more difficult to measure, as they do not touch parts of the car. The best musculoskeletal regions are picked based on a simple ratio between positive and negative counts (#highs/#lows). We picked the neck, shoulders, back, and hips.

**Figure 2:**
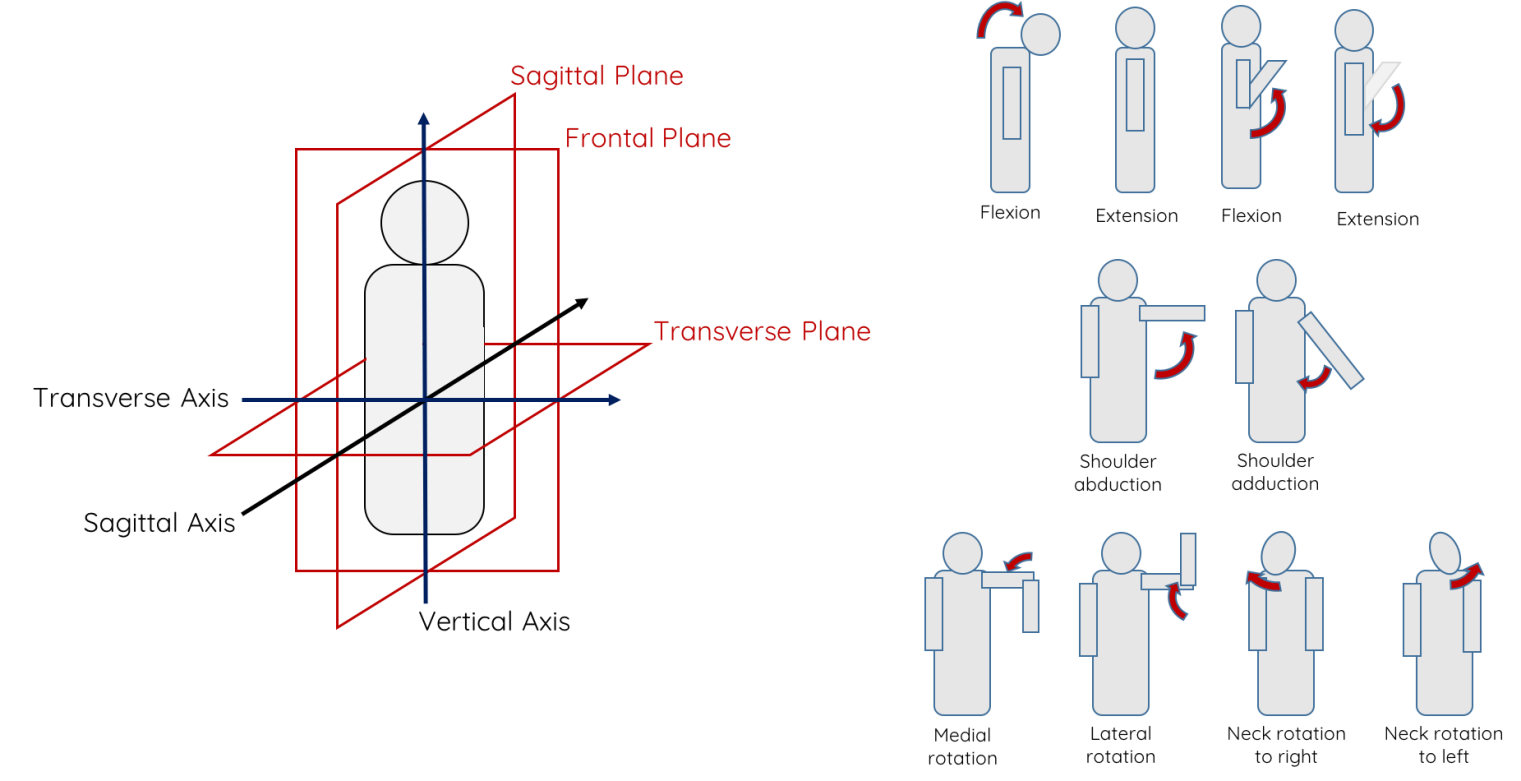
Left: body planes (sagittal, frontal, and transverse) and axes (sagittal, vertical, and transverse). Right: types of movement: extension/flexion, abduction/adduction, and rotation.

To systematically explore a variety of movements, we used a functional anatomical bisection of the body in 3 planes—sagittal, frontal, and vertical—and the axes around which body parts rotate—sagittal, transverse, and vertical [39] (see Figure 2). Movements are described based on these planes and axes. Extension and flexion occur in the sagittal plane about the frontal axis. They measure the increment or decrement of an angle between 2 adjacent body parts. Abduction and adduction occur in the frontal plane about the sagittal axis. They measure the movement away from or toward the vertical axis. Rotation movements occur in the traverse plane and include any twisting motion. We complement these movement descriptions with elevation and depression, the movements in a superior or inferior direction.

#### Simulated Driving

**Figure 3:**
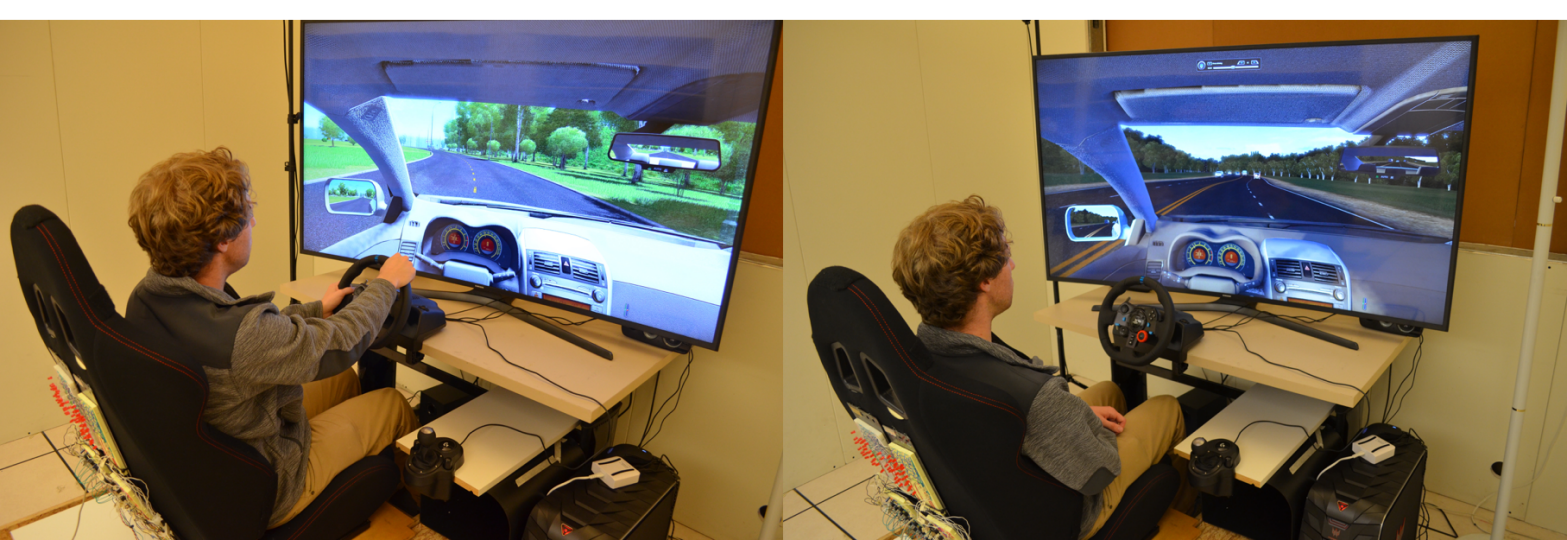
Left: city driving condition. Right: autonomous condition.

Our experiments were developed in a simulator comprising a large 65-inch curved high-definition screen, the vibrotactile chair, and a computer running the City Car Driving software (Forward Development) [40]. We simulated an average automatic transmission car with wheel, pedals, and gear shift controls. People could adjust the seat position, the seat rest angle, and the pedal position. Direct controls to activate signaling lights and parking brake were provided. Figure 3 shows the contrast between manual and autonomous driving modes. Additionally, we evaluated 2 driving conditions: highway (or simple) and city (or complex). The former occurs on a highway with a moderate load of traffic flowing at the nominal maximum speed. The latter occurs in a city downtown area with multiple cars with moderate aggressiveness and a limited number of pedestrians.

#### Vibrotactile Seat

**Figure 4:**
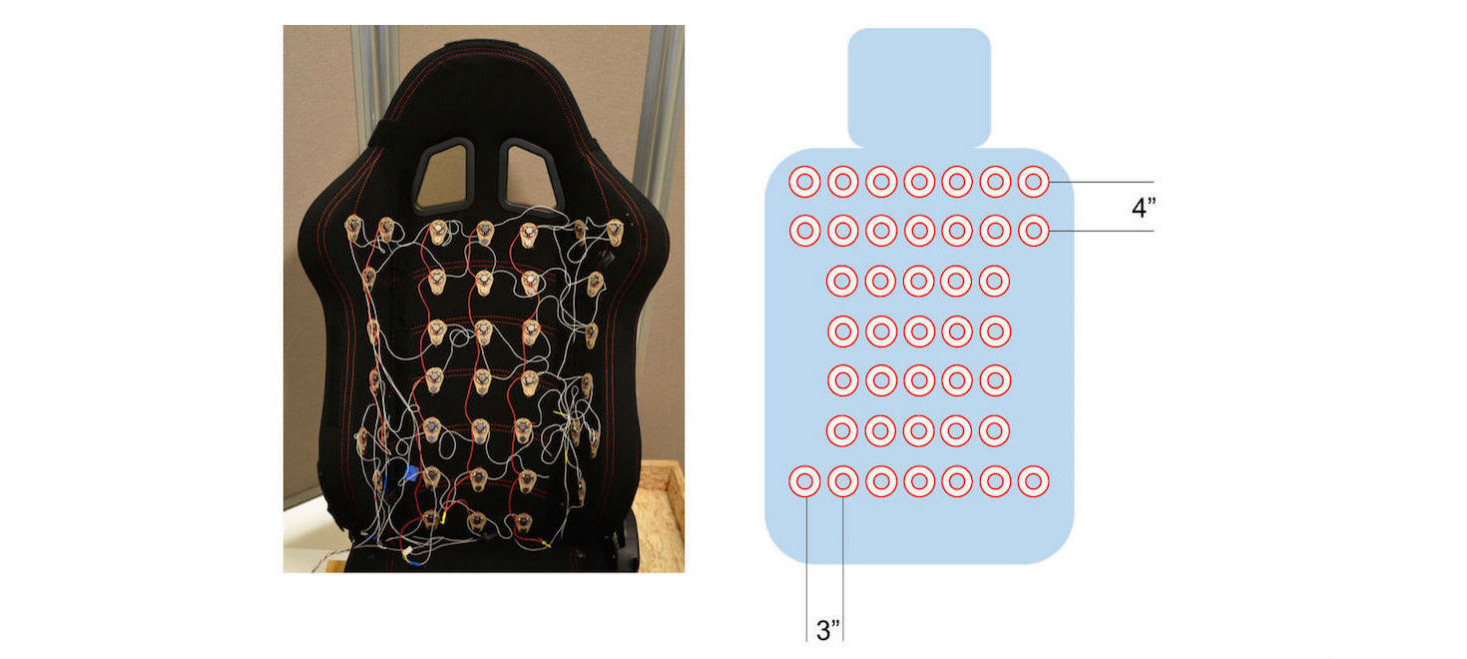
Back rest with 41 vibrotactile motor grid arrangement.

The vibrotactile seat was designed using forty-one 50 dB, 13000±3000 rotations per minute, 2 V to 3.6 V linear resonant actuator vibration motors arranged in a grid covering an area of about 20×26 inches (see Figures 1-4). Motors were 3 inches apart horizontally and 4 inches apart vertically (see Figure 4). The grid area was chosen after testing different body shapes. The separation between motors (3-4 inches) was chosen to guarantee clear 2-point discrimination [41,42] in the back.

**Table 2:** Twelve basic movements. Participants were required to keep eyes on the road only during the manual driving condition.

Movement and body part		Exercise and instruction
**Motor movements**		
	Back and torso	M1—back arch (back flexion/extension): “Arch your back forward and backward.”
		M2—back twist (back vertical rotation): “Twist your torso with your head up. Grip steering wheel for extra support.”
		M3—side stretch (lateral back flexion/extension): “Stretch your back side to side.”
	Head and neck	M4—head turn (head rotation): “Turn your head as if you were checking your blind spots.”
		M5—head bend (head flexion/extension): “Imagine a pigeon head nodding.”
	Shoulder	M6—shoulder lift (shoulder elevation/depression): “Lift and then let drop your shoulders as if you were shrugging.”
		M7—shoulder join (shoulder abduction/adduction): “Join your shoulder blades—could be similar (but not identical) to arching back.”
	Hip	M8—hip sway (hip elevation/depression): “Sway your hips left and right, similar to a dance move.”
Breathing exercises		M9—deep breathing: “Inhale for 4 seconds, hold for 4 seconds, exhale for 4 seconds, hold for 4 seconds.”
		M10—dragon’s breath: “Two vigorous short inhales + one long exhale.”
Visualization, breathing, and posture exercises		M11—deep sigh: “Imagine you have completed a complex task and do a deep sigh (Ahhhh...).”
		M12—loose neck: “Imagine the neck hanging free (such as a bobble head). Move head in all directions so that the back can lengthen and widen.”

### Interactions

#### In-Car Movements

We picked basic movements as building blocks for mindful movement interventions. Table 2 lists 12 different movements (M1-M12), which are aggregated in 3 main groups: motor, breathing, and visualization. Motor movements involve the back (M1, M2, and M3), aimed at performing rotations of the head and neck (M4, M5); the shoulders (M6, M7); and the hips (M8) along the 3 axes, transverse, sagittal, and frontal (Figure 2). Breathing exercises involve deep breathing (M9), which is performed by inhaling through 4 counts, holding the breath for 4 counts, and exhaling for 4 counts, and dragon’s breath (M10), which is performed with 2 high-energy inhales and a vigorous longer exhale. Visualization (M11 and M12) draws inspiration from the Alexander technique [13], which focuses on body posture. We asked people to imagine the completion of a task associated with a deep sigh (M11) or to imagine that the neck is detached from the body and to move the head freely (M12). Participants were required to keep eyes on the road only during the manual driving.

#### Vibrotactile Patterns

**Figure 5:**
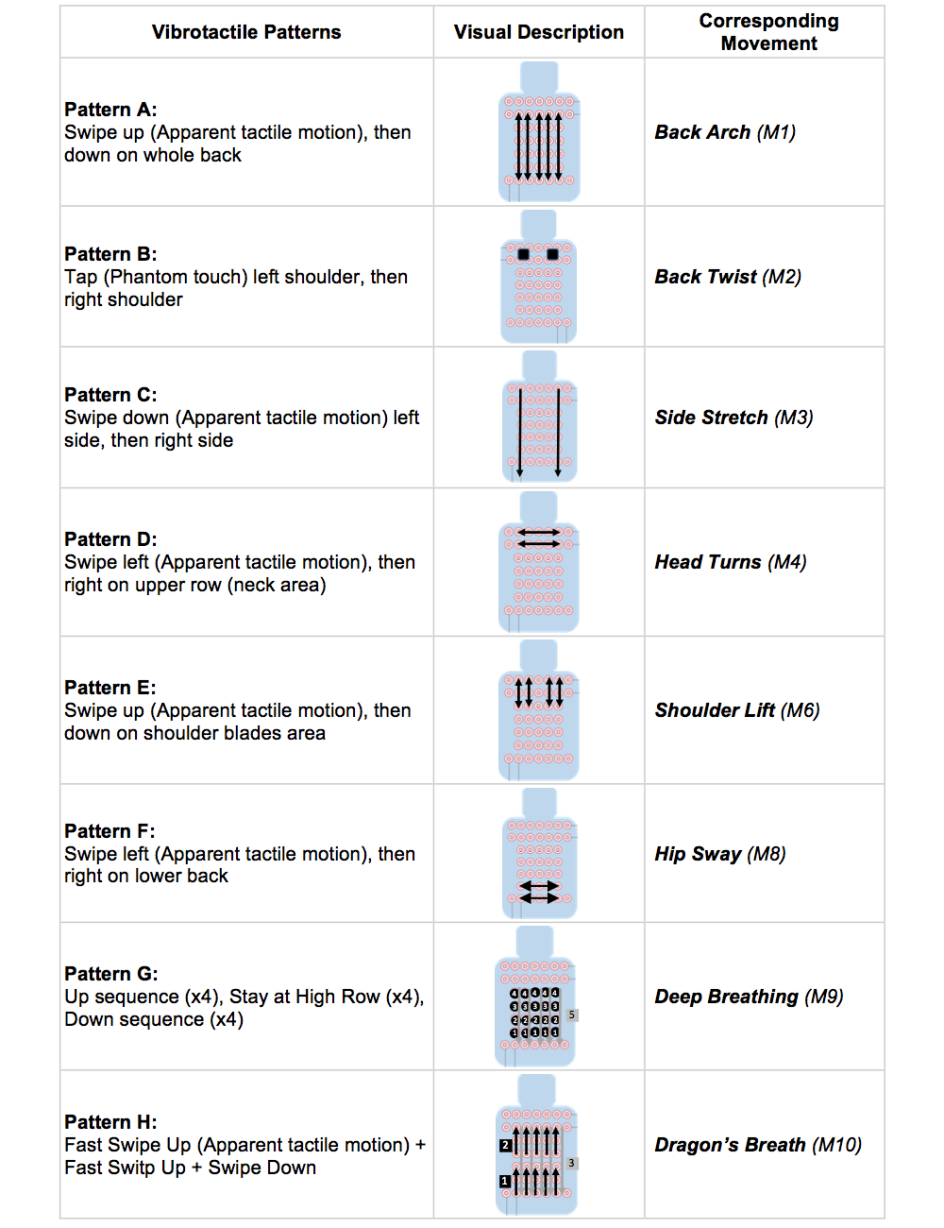
Eight vibrotactile patterns used to study in-car movement elicitation.

We designed a set of 8 vibrotactile patterns that correspond with the same number of in-car movements (see Figure 5) and observed whether participants could map these patterns correctly. Additionally, we inquired whether these patterns prompted them to perform any movements. To design these patterns, we used 2 haptic techniques: apparent tactile motion, which recreates the feeling of a continuous swipe when adjacent motors are activated with an overlapping window of a few milliseconds, and phantom touch, which creates the illusion of a tap, a single contact point when adjacent motors are activated in parallel [29,43].

## Methods

### Participants

We recruited 12 participants: 6 undergraduate students, 3 graduate students, and 3 staff members. The ages of the 4 women and 8 men ranged from 19 to 37 years, with a mean of 26.4 years. Participants reported being more aware of surroundings while driving (mean 8.21) than while being a passenger (mean 5.36). Most participants (10/12) had limited experience with meditation, and half (6/12) practiced some form of breathing-based stress reduction. Most participants (10/12) had no experience with acupressure stress reduction, but most (8/12) had some experience with haptic stimuli from devices such as mobile phones or smart watches.

### Protocol

**Figure 6:**
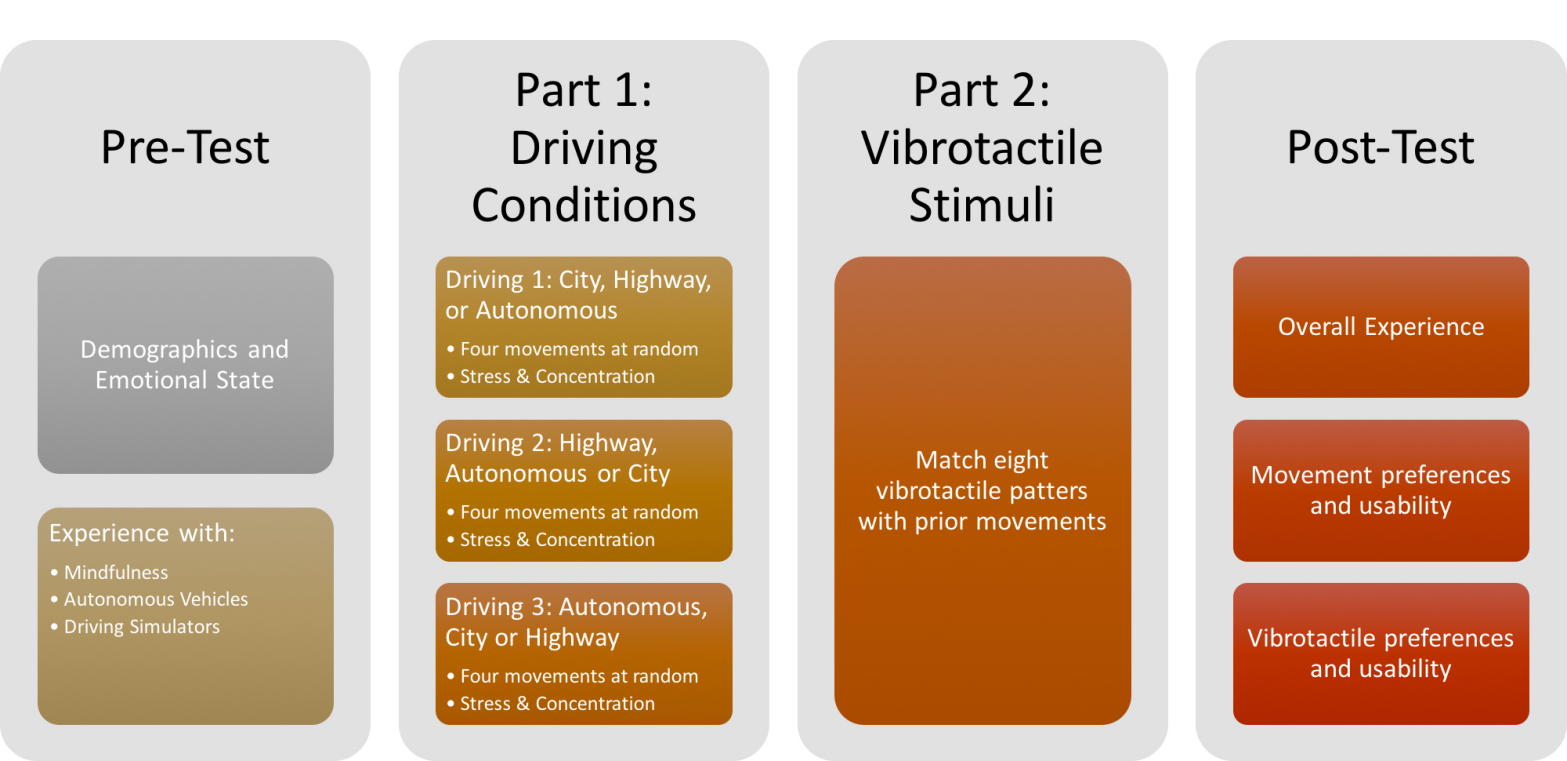
Semistructured assessment protocol consisting of 4 parts: pretest, part 1: movement execution, part 2: movement elicitation, and posttest.

We explored reactions to movement using a semistructured assessment protocol divided in 2 parts: first we explored movement execution during the manual and autonomous driving conditions, and then we explored movement elicitation through a vibrotactile stimulus from the car seat. The experiment lasted on average 60 minutes. We used a pre- and a posttest questionnaire to obtain demographics and information on preferences and usability (see Figure 6).

#### Pretest

Upon arrival, in the preexperiment phase, participants were asked to complete a survey to obtain demographic information and their previous experience with stress management relaxation techniques, meditation, autonomous driving, simulators, acupressure relaxation, and haptic stimuli.

#### Part 1: Driving Conditions

**Figure 7:**
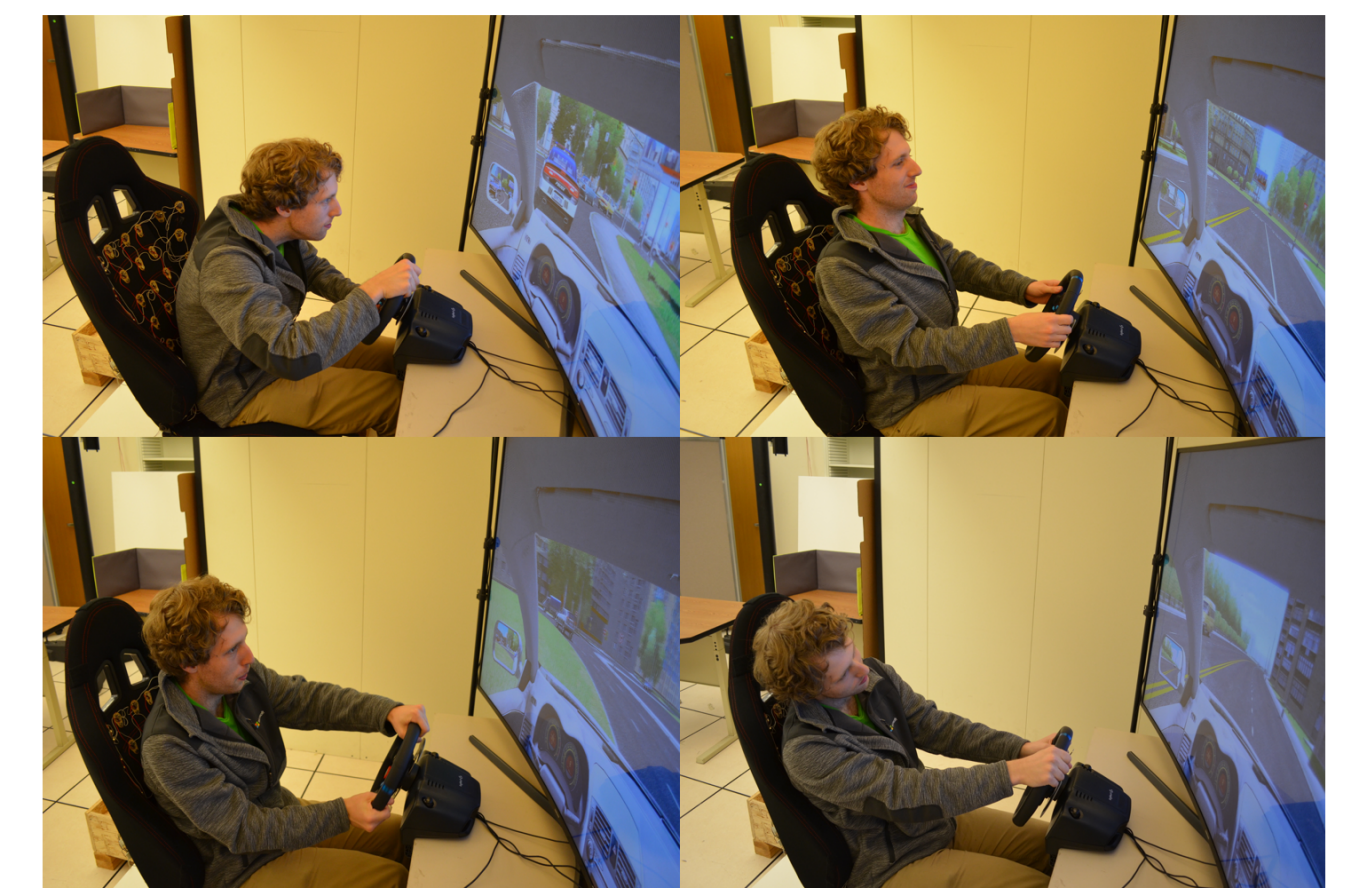
Participant driving in a city condition while performing 4 guided movement instructions: arching back, joining shoulder blades, sighing, and imagining that head was not attached to body.

**Figure 8:**
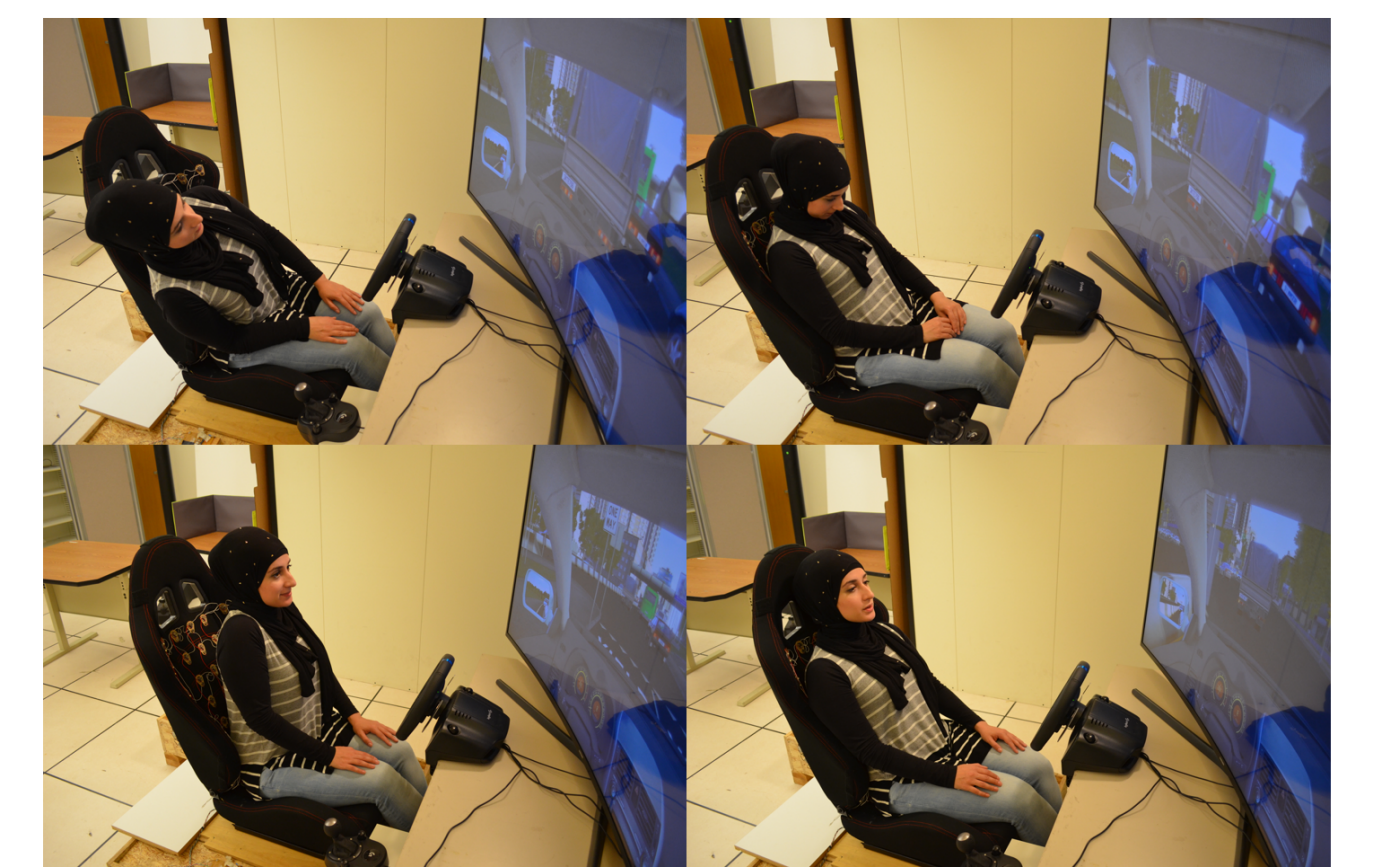
Participant in an autonomous vehicle condition while performing 4 guided movement instructions: twisting back, moving head forward and backward, lifting shoulders (shrugging), and deep breathing.

The participants were assigned randomly (Latin square) to each of the driving conditions: manual + city (complex driving), manual + highway (simple driving) or autonomous + highway and city. Participants verbally received 4 randomized in-car exercises (Table 2) per condition. During each round, we used the thinking-aloud technique to motivate people to talk about their stress and overall experience. After each round, participants were asked to report their favorite movements or exercises from the set and their level of stress and concentration. At the end of the study, they reported their top 3 most desired and least desired movements or exercises. Figure 7 showcases a participant driving in a city responding to movement prompts requesting him to perform the following movements: arch his back (M1), join his shoulder blades (M7), perform a sighing visualization exercise (M11), and imagining that his head was not attached to his body (M12). Figure 8 showcases a participant in the autonomous driving condition responding to prompts to twist her back and torso from side to side (M2), move her head forward and backward (M5), lift her shoulders up and down (M6), and breathe deeply (M9).

#### Part 2: Vibrotactile Stimuli

During the vibrotactile stimuli stage, participants received 8 interactions in randomized order. They responded to the following 2 questions: “Would you agree that this stimulus wants to communicate X interaction?” and “In which driving conditions (city, highway, or autonomous), would you consider using it?”

#### Posttest

During the postexperiment phase, participants responded to the following open-ended questions requesting to describe their experience, outline their preferences, express their reactions to performing movements in the car, and express their reaction to vibrotactile stimuli:

- Can you please describe your experience with the interactions?
- What was your experience like with the haptic seat?
- How did interacting with the haptic seat alter your mental state?
- If you could change anything about the haptic seat, what would you change?
- If you could change the autonomous interaction, what would you change?
- Would you consider driving this car in the future?
- Would you consider using a haptic seat in the future?
- Would you use it as a passenger in a regular car?

We analyzed these answers as well as the videos of thinking-aloud statements using a grounded theory approach [44] aimed at discovering key insights that would guide future research of in-car mindful movement interactions.

## Results

### Overview

We observed a preference for movements that are familiar and less awkward, such as breathing. Some people found unusual movements interesting and believed they may use them in the future. Counterintuitively, for some participants, manual driving on a highway, instead of autonomous driving, seemed to be a condition for earlier adoption of movement-based interventions. It seems that the novelty of a car driving on its own did not allow some people to take full advantage of the movements. Perhaps our simulation of an autonomous car was more aggressive than expected, but, in general, people found themselves monitoring the behavior of the car, in case it made some serious mistake. The city condition seemed to have a cognitive and arousal load that limited the adoption of movement-based interventions.

### Descriptive Statistics

#### Preferred Movements and Exercises

**Figure 9:**
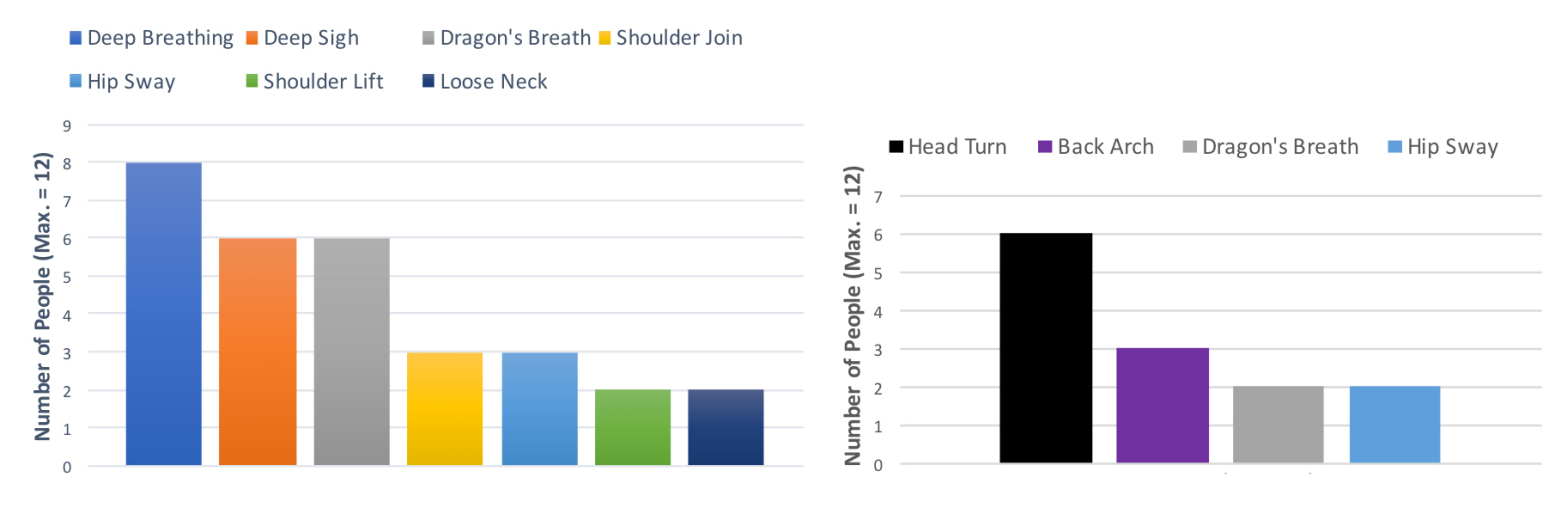
Favorite and disliked exercises.

In this section, we describe the most and least preferred movements and exercises (see Figure 9). Two-thirds of the participants (9/12, 67%) reported that they would do any exercise in autonomous mode. Half of the participants (6/12, 50%) preferred the following breathing exercises: deep breathing (M9), dragon’s breath (M10), and the deep sigh (M11). The rest preferred either the shoulder join (M6) or the hip sway (M8).

[Deep breathing]...Nice. Most natural one. I do that in my meditation.

P5

[Deep breathing]...Not distracting. Helps with boredom.

P1

[Shoulder join]...like this one. It doesn't distract me from driving. It stretches my body.

P12

Participants disliked the head turns (M4) (6/12) because they “couldn’t see the road” (P2). However, one participant found it useful to check the mirrors as well as perform shoulder and torso movements.

It was pleasant. Refresh[ing] alert. Drawing me to look at my mirrors.

P3

Other participants did not like the back arch (M1) (3/12, 25%). One participant (P4) did not like any of the breathing exercises (M9, M10, and M11), and 1 (P8) found the dragon’s breath (M10) “strange.” Two participants (P9 and P12) disliked the hip sway (M8), as it messed with the use of the pedals.

**Figure 10:**
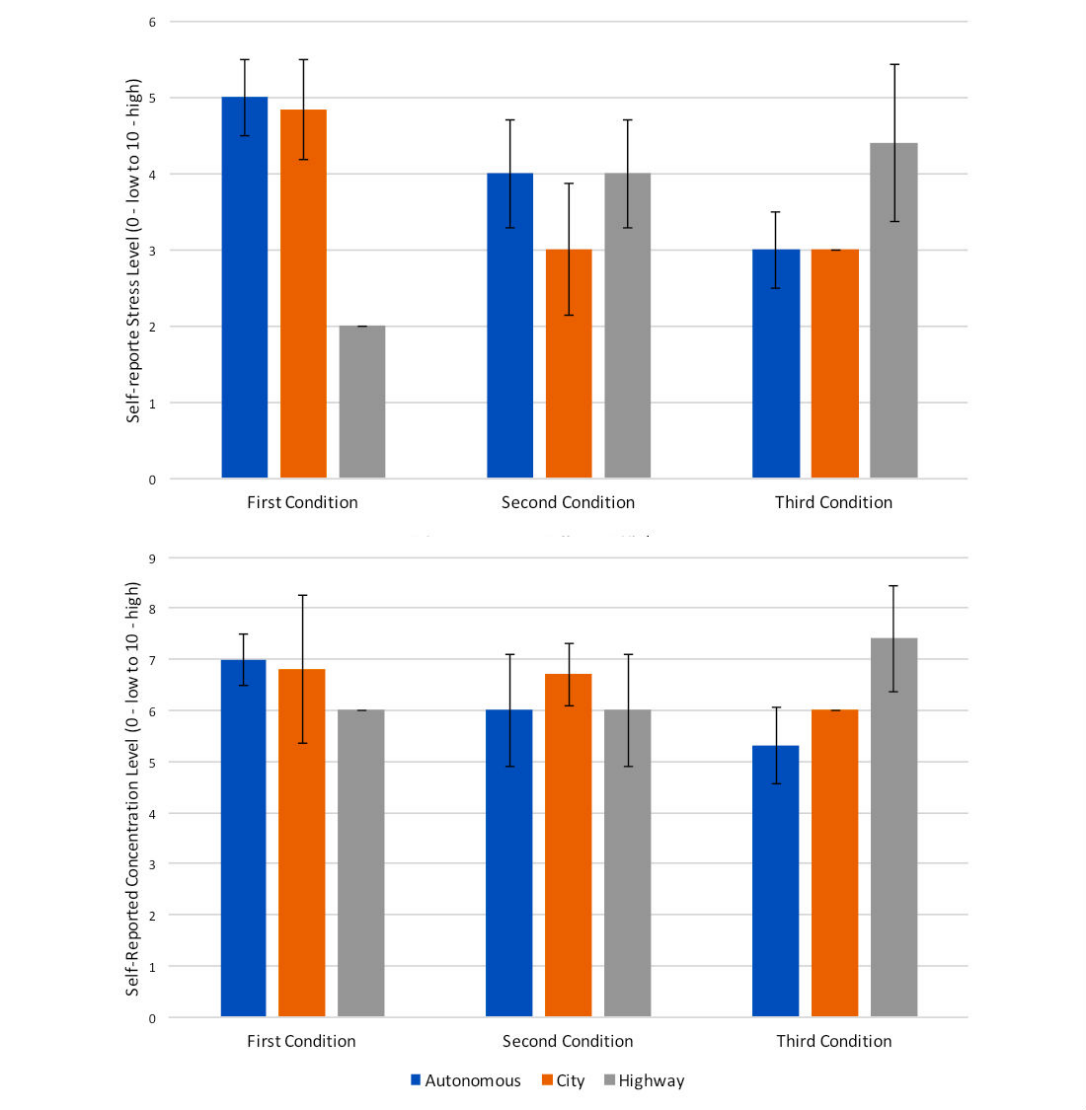
Stress and concentration self-reported metrics for each driving condition, city, highway, and autonomous (city and highway), versus the order in which they were presented in the study. Error bars represent standard error.

#### Perceived Stress and Motion Sickness

As expected, participants reported differences in the perceived stress for each of the driving conditions, and the order seemed to influence this perception (see Figure 10). In the autonomous and city conditions, there was a tendency to have lower stress and lower concentration when these conditions were second or third. This could be because the benefits of not driving are perceived only after people get used to the simulator or the driving exercises. This could indicate a potential interaction effect between complexity and novelty, which should be studied in a controlled experiment. In the highway or simple condition, the tendency is reversed. Here it seems that people were more stressed and had higher concentration when this condition appeared last in the study. Perhaps this tendency can be explained by sheer fatigue. In summary, we believe that novelty of the simulator, experiment, or driving condition could affect complex or new (autonomous) driving conditions, whereas fatigue could play a role in a simpler or less engaging condition.

People reported the autonomous condition to be more aggressive than expected. Some people described it as if in some cases the car were about to crash or make abrupt lane changes.

I cannot keep my eyes on the road, I don't trust the car.

P2

**Figure 11:**
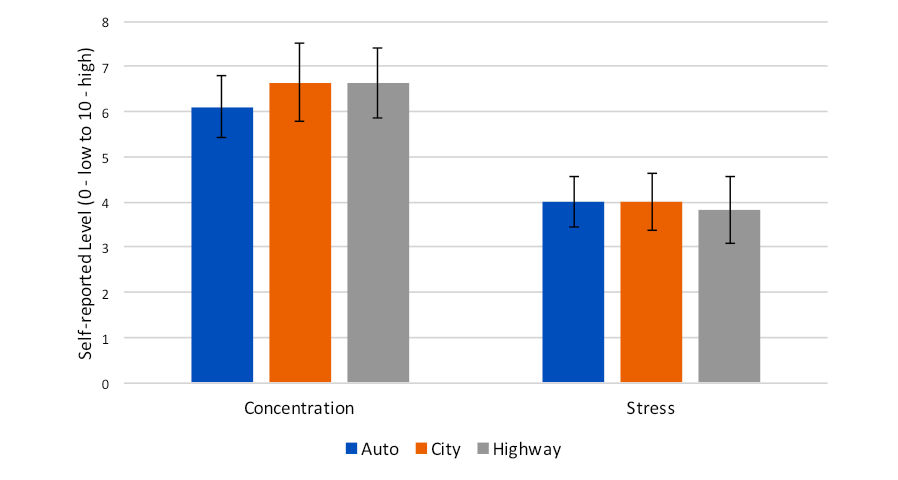
Perceived average concentration and stress. Error bars represent standard error.

Despite these differences, on average stress and concentration seemed to be comparable across all conditions (see Figure 11). These self-reported perception metrics contrast with personal preferences, as described in later subsections. Retrospectively, however, people found the system stressful. Some (4/12, 33%) found the system stressful in both conditions, and some (3/12, 25%) found it only stressful during the manual condition. However, on average people were not affected by the simulator. The total raw score (4.41) was below the average score (7.12) for a virtual reality experience with 157 patients reported by Bouchard et al [45]. Similarly, the nausea average score (1.91) and the oculomotor change (2.5) were both below the similar benchmark metrics [45] for nausea (3.51) and oculomotor change (2.86). People did not complain of motion sickness, although some reported fatigue.

### Participant Comments

#### Movements Already Used in Car Situations

Some participants reported using some of the movements already in their daily driving experience. One participant (P12) performed back arching (M1) when fatigued; 3 other participants did different movements to reduce stress. Participant 8 did back twists (M2) and shoulder lifts (M6), participant 3 commonly performed side stretches (M3) and shoulder lifts (M6), and participant 10 did shoulder joins (M5). One participant (P10) practiced meditation and would like to do it in the car, whereas another (P5) practiced deep breathing (M9) but preferred not to do it while driving, as he felt it could be distracting.

#### Best Time to Do Movements and Exercises

During manual conditions, the best option to move was during a simple driving condition (highway) in straight paths or while stationary—for example, in a traffic jam or at a stop light.

I would do the sigh breath on the highway or at red lights because otherwise it consumes my concentration.

P5

I won’t stretch my hips while driving. Maybe in auto and at a red light.

P6

It is okay. I don't feel secure doing this [back arch (M1)] while driving. [Distracting?] Yes. [Better when stopping?] Yes, so I am not distracted.

P115

In the city condition, drivers slowed down and waited for a straight path to avoid traffic or pedestrians. One participant performing deep breathing (M9) mentioned that it helped him keep his concentration.

It [deep breathing] is easiest so far. It is calming. I can keep my concentration.

P3

Three participants mentioned that a deep sigh (M11) would be interesting if it was cued just after an incident has occurred.

We observed a paradoxical response with respect to the autonomous condition. When asked about their experience with the movements, several participants found the autonomous condition a good time to do exercises. However, half the people (6/12, 50%) described the aggressive nature of the autonomous car as stressful. For instance, although it was not required, a few participants reported feeling stressed trying to keep an eye on the road, precluding them from doing exercises.

The autonomous/last drive was the nicest, but most heart-wrenching. Less responsibility over driving feels better.

P1

Context could play an important role for some interactions. For example, some people reported feeling self-conscious while doing unconventional movements that felt silly, whereas others noted that adding music to an otherwise unconventional movement could help.

[Back arch]...Weird, I cannot see. People will think I am in distress.

P9

Movements that reestablished symmetry and balance were appreciated.

[Deep breathing]...Reminds me to sit upright...I would like to be reminded to sit upright.

P2

However, 1 user refused to perform the head (M4) and neck (M12) movements while switching lanes or at a busy intersection.

[Head bend]...It’s a busy intersection and this is a distraction.

P7

[Loose neck]...Impossible to do that and drive. I cannot stay in the lane. Stressful.

P7

#### Movements That Interfere with Perceived Driving Performance

We evaluated how movements affected the participant’s perception of driving performance. We focused on movements that seemed to interfere with their regular behaviors while driving, such as steering, visual attention, foot pressure, or other body-related interferences to a comfortable and safe driving experience. Most participants (10/12, 83%) felt that lateral movements would most likely interfere with visual coordination and steering because they made them move their arms and legs from side to side. For example, back twist (M2) led 1 participant (P6) to a complete stop. Side stretch (M3) affected the steering and reduced their field of view.

[Side stretch]...I cannot drive straight.

P6

I dislike the side stretch...because I couldn’t look at the mirrors.

P3

Lateral exercises that involved movements affecting the legs, for example, hip swaying (M8), affected the foot pressure on the pedals and steering.

Everything that moves the lower body affects the pedals.

P9

Strange, it messes with my gas pedal.

P12

Hard to move without releasing pressure on the pedal and moving the steering wheel.

P2

Although nonlateral movements were less likely to interfere with steering, 1 participant felt uncomfortable as the back arch (M1) did not allow him to constantly monitor his surroundings:

Strange to do in a car. You lose your overview of the mirrors.

P4

Movements such as bending the head back and forth (M5) or sideways (M6) were reported by some (P6, P11, and P12) as distracting, affecting their use of glasses and in some cases even inducing some motion sickness.

Cannot see the road, my glasses shift off my eyes.

P12

#### Movements That Complement Perceived Driving Performance

Whereas lateral exercises tended to interfere with driving, forward and backward and vertical motions were reported by most (9/12, 75%) to have less impact on vision and steering.

Forward [and] backward movements and deep breathing don’t affect my control of the wheel.

P2

However, some people complained about the size of the seat, which limited movement.

The seat is tight, I do not feel I am moving right.

P2

It feels good. Strange because the seat is enclosing me when I go back. I do something similar in my car.

P12

Some participants (3/12, 25%) felt that lateral exercises were more appropriate when stopped at an intersection because not only was steering less of an issue but the lateral movements helped enhance their awareness of surroundings.

In contrast to motor exercises (M1-M8), deep breathing (M9) was reported by most participants (8/12, 67%) to work well because it did not physically affect steering and thus could be done even while turning. A few (3/12, 25%) found it challenging to keep count of their breaths while driving because it involved mental effort.

I feel relaxed. Doing the exercises made me remember to breathe slowly.

P2

I needed to put a lot of mental focus for the movements, maybe because I am not used to them. I will probably not do the breathing in a busy road like this though.

P3

Dragon’s breath (M10) was considered by 2 users (P9 and P10) an energizing exercise that could be used to fight fatigue or boredom.

#### Exercises That Are Relaxing

Deep breathing was considered by many participants (9/12, 75%) to be relaxing. People also found it relaxing to do head and neck (M4/M5) and shoulder (M6/M7) movements because they loosened spots that were often stiff or sore.

Rolling shoulders is more soothing than dropping. I do it naturally during my commute, when I feel stiff.

P2

Almost half of the people (5/12, 42%) found the backward back arch (M1) (“antislouching”) and hip sway (M8) relaxing and particularly useful during long drives. In contrast, the forward back arch (M1) felt unnecessary because it resembled slouching habits.

I already do this [hip swaying] after a long trip.

P9

[Hip sway]...wants to make me shoot my hips. I appreciate it in the highway condition; it reminds me to move cause I’ve been sitting for a long time.

P2

#### Coherence of Vibrotactile Patterns

People could match 52% (50/96) of the vibrotactile stimuli to their intended movements either immediately or after 1 guess (see Table 3). Almost 1 in 5 participants (18/96, 19%) explicitly reported that the patterns made sense whereas only 9% (9/96) reported that they didn’t make sense. Three movements were guessed correctly by more than half of the participants: back twist (M2) (7/12, 58%), side stretching (M3) (8/12, 67%), and deep breathing (M9) (7/12, 58%) (see Table 3). In general, people found these movements relaxing, good for stretching, or not distracting. In contrast, 2 movements were guess correctly by less than half of the participants: dragon’s breath (M10) (4/12, 33%) and back arch (M1) (5/12, 42%). The others reported being clueless on what the vibration was trying to tell them or felt the pattern was better mapped to an alternative movement. One of them reported that the vibration stimuli should have less intensity (P5).

The reasons why people did not guess correctly were varied (see Table 3, column 3). Some people guessed the intention of the vibrotactile pattern (such as sideways movement) but did not know which part of the body to move; others, despite liking the sensation of the patterns, mapped them incorrectly. Some complained that vibration should simply not be used as a stimulus. One person (P9) could hear the vibration because she was too short, and another (P3) found the vibrations signaling movement of the hips too high. Some found that patterns were similar and that made it impossible to distinguish them, whereas others just could not guess the intention and preferred to use it as a breathing guidance.

#### Usability of Vibrotactile Triggers

Almost all participants (10/12, 83%) found the idea of at least 1 vibrotactile pattern appealing for autonomous or highway conditions.

It depends on the driving mode. I would like to do my stretchy movements in auto. I don't feel like I am risking my personal safety in auto.

P1

Most people (11/12, 92%) did not express specific interest in using vibrotactile cues during the city (complex) driving mode. They believed using them might add an unnecessary additional cognitive load. However, some suggested performing deep breathing or shoulder movements while sitting at a red (traffic) light. One participant reported that the vibrotactile signal would make her more alert and that might be helpful to remind her to check the mirrors.

[Head sideways pattern]...It was pleasant…[a] refresh[ing] alert. Drawing my attention to look at my mirrors.

P3

Most people (8/12, 67%) found deep breathing (M9) either relaxing or pleasurable. Some may have preferred a slower pace.

[Deep breathing]...Pleasing...I would like a slow vibe up my spine.

P3

However, 1 of the 8 suggested instead using air flow (coming from the air conditioning system) to signal when to breathe.

Vibration is about movement not breathing for me. Use air flow to signify breathing instead of vibrations.

P11

The participants who did not like the deep breathing pattern thought that the vibrations were either too strong or that they signaled movement rather than breathing.

I don't like it. It is vibrating my internal organs.

P2

Vibrations that go up and down signify movement. Breathing vibes should be local.

P12

Although all users thought that doing some exercises in the car would be useful, only half (6/12, 50%) reported that they would use a haptic seat in the future in its current state. Only one-third (4/12, 33%) would use it frequently. Others (5/12, 42%) would use it if the following improvements were made: make the seat a different size (P2 and P8), make the vibration stronger (P4) or weaker (P12), improve the vibration patterns (P6), add a massage option (P9), and detect the right moment to use the vibrations (P12).

**Table 3:** Matching vibrotactile patterns and in-car movements.

Vibrotactile pattern		Correct	Reasons for guessing incorrectly
**Pattern B: tap left shoulder, then right shoulder.**		8/12	“...it is telling me to move sideways but head, shoulder, back [?]” [P11]
	M2—back twist		
**Pattern C: swipe down left shoulder, then right shoulder.**		8/12	“Like it. Hip twist.” [P3]
	M3—side stretch		
**Pattern G: swipe up, hold, swipe down, hold.**		7/12	“Vibration is about movement not breathing for me.” [P11]
	M9—deep breathing		
**Pattern D: swipe left, then right on neck area.**		6/12	“I don't feel it but I can hear it.” [P9]
	M4—head turn		
**Pattern E: swipe up and down shoulders.**		6/12	“...It is very similar to others in that area.” [P5]
	M6—shoulder lift		
**Pattern F: swipe left, then right on lower back area.**		6/12	“For hip twist put it lower...” [P3]
	M8—hip sway		
**Pattern A: swipe up, then down on whole back.**		5/12	“Shoulder shrug? I will just do breathing.” [P9]
	M1—back arch		
**Pattern H: swipe up-up-down.**		4/12	“Too many pulses for dragon’s breath.” [P3]
	M10—dragon's breath		

## Discussion

### Principal Findings

This exploration of in-car movement reveals the potential for interventions that take advantage of movement and breathing techniques. Movement-based interactions should adapt to contextual cues, participant preferences, and road conditions. Specifically, movements that interfere with steering and pedal function, such as lateral flexions and extensions, should be avoided or only used during autonomous driving or when stopped. In contrast, neck or shoulder movements appeared to offer relief. Some even aided the driver by increasing lateral awareness at a stop light or breathing and sighing mindfully after an incident. Deep breathing may be a particularly suitable exercise because of its capacity to relieve stress without physically interfering with steering. Haptic patterns that are well modulated may off-load the cognitive burden of keeping count during deep breathing. On the other hand, some movements may require coaching to be safe during driving. For example, the lateral flexion and extension, although helpful in stretching the back and torso, may not aid driving. Participants should pick their preferred movement and how often it is prompted. New or unusual movements should be introduced gradually.

Overall, autonomous driving seems to be an opportunity to adopt more movement-based interventions in the car. However, movements should allow people to see the road, at least until they feel confidence in the automation system.

### Design Considerations

#### Detecting Driving Modes Is Fundamental for a Safe System

**Figure 12:**
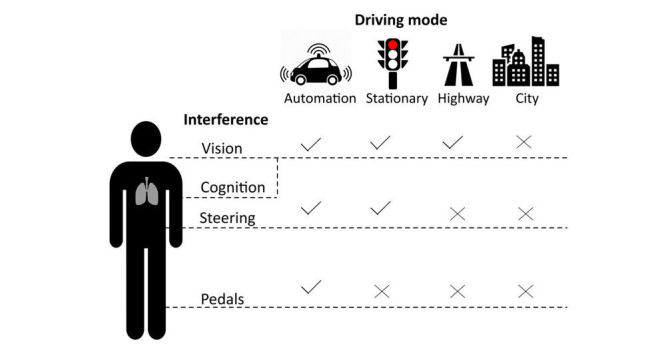
Interferences caused by movement-based exercises during different driving modes.

It is essential to consider the participant’s driving mode (autonomous vs manual) and situation (highway vs city) before designing any movement-based interventions. Different movements can interfere with the 3 main body functions needed for proper driver performance: vision, steering, and pedals. Figure 12 contrasts these functions with driving conditions. In general, participants did not want to perform any movement-based exercises while driving in the city. Given the number of stimuli, even mild movements such as deep breathing or shoulder lifts could cause alterations in the body functions. On the highway, participants did not want to perform any movements that impaired their steering or pedal functions. They would consider movements that had a small effect in their vision, such as deep breathing and shoulder movements. In stationary situations, such as being parked or at red lights, people were willing to move the torso or do stretches, but they preferred not to do movements that affected their pedals. All participants agreed that performing any movements in an autonomous car they were familiar with would feel safe, keep them alert, and could improve their well-being. In all cases, participants must have control over the system and should be able to activate or deactivate it quickly.

We observed a couple of participants ignoring the instruction to perform a movement if they were in tough situations such as changing lanes or crossing an intersection. Some participants also reported that haptic feedback while they were in the complex driving condition (city) could be distracting. Thus, a safe system must be able to detect these situations, for example, by measuring the participant’s physiological signals or by using eye trackers and road cameras. Alternatively, the system could be activated only while the car is stationary, at a red light, or during a traffic jam (navigation apps such as Waze [46] detect when a car is stationary and display visual advertisements on the participant’s screen).

#### Vibrotactile Stimuli Should Be Clear and Customizable

The intensity, location, and pattern characteristics of the vibrotactile stimuli should be configurable. Haptic signals must be strong enough to be discernible from external vibrations and subtle enough not to distract the participant. Body types must be considered for the layout of the motors. In our study, participants who felt shoulder vibrations too high or too low on their torso were rather confused. Patterns cueing motor movements should be clearly distinguishable from breathing ones. One participant suggested fixing the breathing pattern to 1 body location and having motor movements signal directionality with more ample patterns.

Patterns that guide movements should adapt to baseline conditions. For example, some people indicated a desire for a slower breathing pattern. One option would be to adapt the pace of breathing to the baseline (at rest) breathing rate. The breathing rate could be measured during a stationary condition before the drive. In any case, the pace should be overridable by the user.

Finally, vibrotactile stimuli should not be the only mechanism to stimulate movement. Pressure, air flow, or position could play a role. A multimodal interaction that mixes voice commands, sounds, and lights could perhaps be even more relevant.

#### Nudging and Feedback Are Necessary for Correct Execution

Some participants perceived vibrotactile patterns as mere notifications rather than movement guides. Some waited until a vibration pattern was completed to identify and perform the target exercise. To mitigate this, a haptic seat should have a simple nudging system. Scaling the system to better match the range of motion and timing the vibrations to match the duration of a movement will improve how participants follow and execute an instruction.

Feedback, in haptic, visual, or auditory form, may also be necessary for correct execution, especially while the participant is learning to use the system. Sensors such as depth cameras, pressure mats, and breath sensors can be used to evaluate participant movement and provide feedback to the participant and designer.

#### Practitioners Are More Likely to Accept Mindful Interventions

Participants who performed meditation or had previous experience with breathing techniques or stretching were more positive about certain movements during the study. Some expressed interest in having a vibration system that would remind them to breathe and stretch more regularly. On the other hand, nonpractitioners questioned the benefits of these movements and instead expressed their desire to use the haptic chair for massage. It is important in future systems to consider both types of participants. Participants who are unaware of the benefit of mindful movements are less likely to adopt a movement-based mindfulness system. A simple introductory set of interventions could be helpful in this case.

#### Movements Must Comply With the Available Space

Driver movement is limited by the physical constraints of the car, such as the steering wheel, the car seat shape, the door, and the seat belt. For example, when participants attempted to stretch their torso side to side they felt enclosed in the seat and had to pull their back away from the chair to avoid hitting the seat’s edges. Similarly, participants felt physically restrained while performing hip sways and back arches. Motor movements in the car should not require a large space. Participants feel uncomfortable and are less willing to perform a movement if they cannot perform it correctly. Even though the seat belt is not designed to constrain the driver’s movement, sudden movements can activate the belt safety functionality, holding the participant back. Movements that require people to press against the seat, such as those practiced in yoga or Feldenkrais, could be beneficial.

#### Movement Execution Is Influenced by Social Perception

Participants reported that most movements felt natural to them, and many already stretch their back and shoulders during their commute. However, movements such as the “bobble head” were socially awkward. This may preclude people from doing exercises even while at a red light, because people are more aware of the surrounding drivers. We suggest evaluating the choreographic aesthetics of the movements, adding a complementary stimulus such as music or providing a technical solution such as shading the windows for privacy.

### Future Work

The aim of this paper was to incite and guide researchers and designers studying mindful movements to design and evaluate novel interventions for in-car practice for commuters. We have started engagement with instructors who have responded favorably to our request and with whom we plan to codesign specific in-car interventions for commuters. We aim to validate the efficacy of the interventions in simulator studies and a realistic environment (ie, in a real car). On the basis of the results learned from this study, we plan to focus on the following interventions.

#### Guided Movement

We plan to work with yoga instructors and chiropractors to explore the use of the car seat space and haptic stimulation to design mindful movement interventions for commuters. Initial interactions reveal two types of movement sequences: subtle movement that could be used to deliver immediate results during the highway (simple) driving condition and higher range movement that can be used in the autonomous driving condition. We intend to use a pressure sensor and depth cameras to capture the movement and provide a closed-loop mechanism to enhance the learning process of drivers. Early observations show an opportunity to reduce range of motion constraints by performing movements that require the participant to press against the seat instead of freely moving any joint.

#### Guided Breathing

We are currently using breathing rate sensors to detect the participant’s rate and adapt the vibrotactile stimuli to the participant’s breathing pace. We plan to explore short and long sequences that can be used while waiting at a red light, cruising on a highway, or being stationary. We hope to explore the effects of deep and shallow breathing. The former could be used to reduce stress, whereas the latter could be used to recover from fatigue.

### Conclusions

In this paper, we introduce the basis for the design of novel movement-based mindful exercises. Through report and observation, we establish preliminary design considerations for interactions that leverage mindfulness through movement and breathing techniques. We describe the use of vibrotactile stimuli produced by the car seat to guide and nudge these movements. Implications from our study provide insights into the movements that complement and interfere with driving and the best way to communicate these movements through haptics.
